# Overcoming chemoresistance in prostate cancer with Chinese medicine Tripterygium wilfordii via multiple mechanisms

**DOI:** 10.18632/oncotarget.10868

**Published:** 2016-07-28

**Authors:** Zhijun Wang, Ranadheer Ravula, Leming Shi, Yunjie Song, Steven Yeung, Mandy Liu, Bernard Lau, Jijun Hao, Jeffrey Wang, Christopher Wai Kei Lam, Moses Sing Sum Chow, Ying Huang

**Affiliations:** ^1^ Center for Advancement of Drug Research and Evaluation, College of Pharmacy, Western University of Health Sciences, Pomona, CA, USA; ^2^ Department of Pharmaceutical Sciences, College of Pharmacy, Western University of Health Sciences, Pomona, CA, USA; ^3^ Center for Pharmacogenomics, State Key Laboratory of Genetic Engineering and MOE Key Laboratory of Contemporary Anthropology, Schools of Life Sciences and Pharmacy, Fudan University, Shanghai, China; ^4^ College of Veterinary Medicine, Western University of Health Sciences, Pomona, CA, USA; ^5^ State Key Laboratory of Quality Research in Chinese Medicine, Macau Institute for Applied Research in Medicine and Health, Macau University of Science and Technology, Taipa, Macau

**Keywords:** chemotherapy, resistance, Chinese medicine, Tripterygium wilfordii, prostate cancer

## Abstract

A leading cause of cancer chemotherapy failure is chemoresistance, which often involves multiple mechanisms. Chinese medicines (CM) usually contain multiple components which could potentially target many mechanisms simultaneously and may offer an advantage over single compounds that target one mechanism at a time. The purpose of this study was to investigate the chemosensitizing effect (CE) of a specific CM, *Tripterygium wilfordii* (TW), on prostate cancer cells resistant to docetaxel (Dtx) and identify the potential mechanisms. The CE of TW (in combination with Dtx) was evaluated in two Dtx resistant prostate cancer cell lines (PC3-TxR and DU145-TxR) and the efficacy of the combination for resistant PC3-TxR tumor was investigated using a xenograft mouse model. For mechanistic study, the inhibitory effect of TW on P-glycoprotein activity was assessed. In addition, novel gene targets of TW were identified using DNA microarray and quantitative PCR. Results showed that TW induced a CE of 8 and >38 folds in PC3-TxR and DU145-TxR cells, respectively with Dtx IC_50_ reversed back to that of the sensitive parent cells. An optimum dose of TW+Dtx significantly retarded tumor growth in mice compared to TW or Dtx alone. TW inhibited P-glycoprotein activity and induced a significant gene expression changes in genes related to angiogenesis, cell cycle regulation and differentiation. Our *in vitro* and *in vivo* studies demonstrate that TW in combination with Dtx was able to overcome the chemoresistance and suppress resistant prostate tumor growth via multi-mechanisms.

## INTRODUCTION

It is estimated that 13 million Americans are living with cancer and cancer mortality remains a major health concern (www.cancer.gov/statistics and http://david.abcc.ncifcrf.gov). One important reason for such high mortality is chemoresistance resulting in therapeutic failure. Clinically, chemoresistance is believed to be responsible for treatment failure in over 90% of patients with metastatic cancer [1].

It is well known that all types of cancer can develop resistance to commonly used chemotherapeutic drugs via multiple mechanisms [2]. One of the well-known mechanisms is the overexpression of the efflux transporters such as ATP-binding cassette superfamily members including P-glycoprotein (P-gp) and multidrug resistance-associated protein 1 (MRP1). As a result, many anticancer drugs can be pumped out from intracellular to extracellular sites [3, 4]. Numerous inhibitors of P-gp have been consequently developed with the intention of restoring the sensitization of cancer cells to the chemotherapeutic agent [5–7]. However, none of the P-gp inhibitors have so far been proven clinically successful due to co-existence of other mechanisms [8–10]. Therefore, using a single compound to target a single mechanism of chemoresistance is not likely to be successful.

Chinese medicines (CM) have been used for thousands of years to restore imbalance of body functions due to diseases, possibly by targeting multiple mechanisms based on the holistic theory [11, 12]. Many CM have been reported to contain multiple compounds with significant anticancer activity [13], e.g., celastrol and triptolide from *Tripterygium wilfordii* exhibited significant inhibition of prostate cancer cells [14–17]. Since the holistic theory of CM is based on the interactions of bioactive components in the body to restore balance of function, this may also explain the efficacy of CM in reversal of chemoresistance [13]. Our overall hypothesis is that CM are able to offer advantages over single chemical compounds by targeting multiple mechanisms and thereby restoring the balance of body functions

Based on the above premise as well as a previous positive report of CM for prostate cancer [18], we initiated screening of CM for potential chemosensitizing effect in prostate cancer cells resistant to docetaxel (Dtx, which is the drug of choice for metastatic castrate resistant prostate cancer, or mCRPC) [19]. One CM, *Tripterygium wilfordii* (TW), or Lei Gong Teng, also named Thunder God Vine [20, 21], was found to be especially effective in sensitizing prostate cancer cells resistant to Dtx. Based on the positive results of our *in vitro* study, an animal xenograft tumor model was subsequently carried out to verify the chemosensitizing effect of TW in combination with Dtx as well as identified several mechanisms associated with such chemosensitizing effect using P-gp accumulation and gene expression profiling studies [22, 23].

## RESULTS

### Chemosensitizing effect of TW

**Figure 1:**
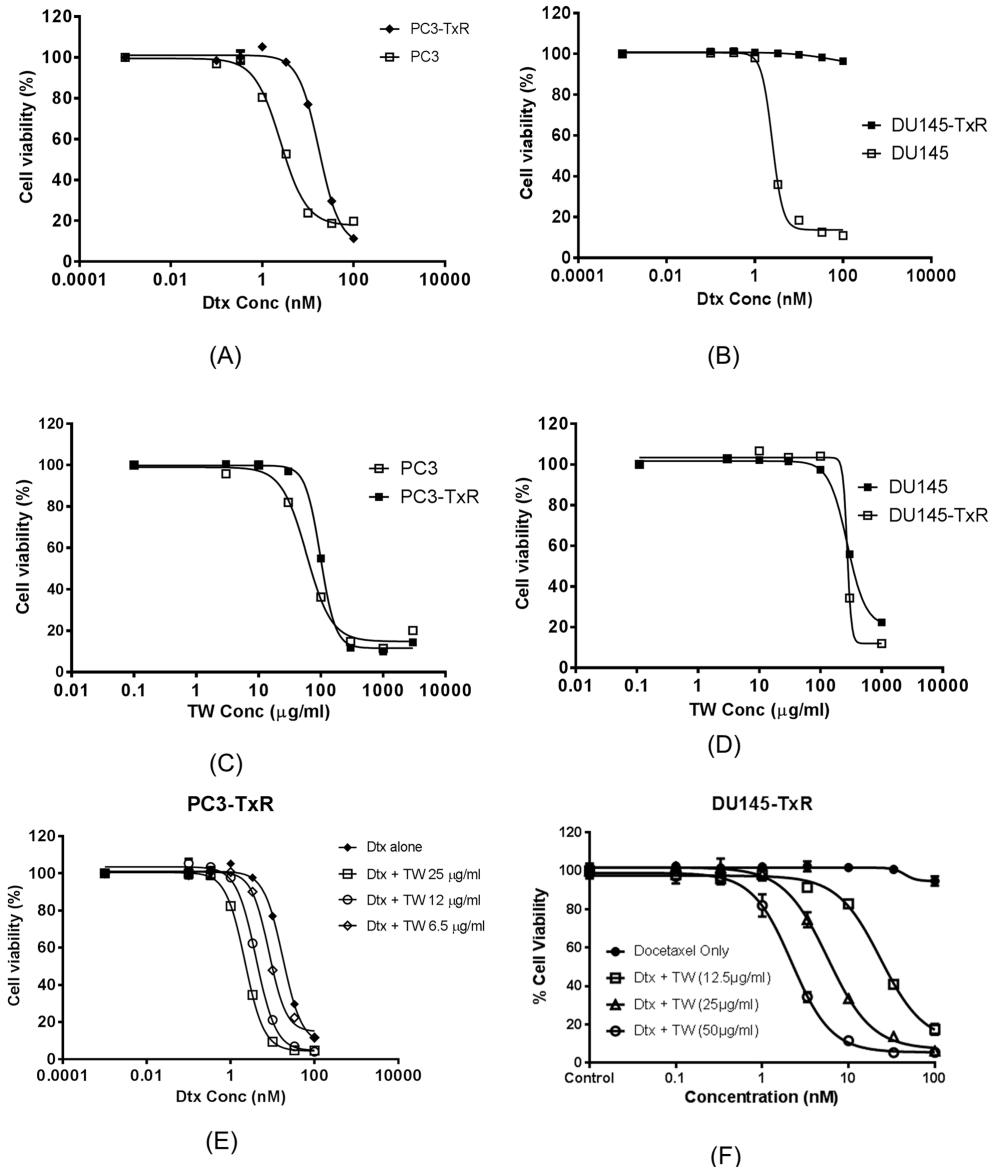
The cell viability (measured from triplicate samples) of prostate cancer cell line (PC3, DU145) and their Dtx resistant cell lines treated with Dtx, TW or in their combination **A.** The cytotoxicity of Dtx on PC3 and PC3-TxR (IC_50_ 2.41 ± 0.12 and 19.7 ± 3.3 nM respectively); **B.** The cytotoxicity of Dtx on DU145 and DU145-TxR cells by Dtx (IC_50_ 2.61 ± 1.3 nM or IC_50_ of >100 nM respectively); **C.** The cytotoxicity of TW on PC3 and PC3-TxR (IC_50_ 46.3 and 48.3 μg/ml respectively); **D.** The cytotoxicity of TW on DU145 and DU145-TxR (IC_50_ 0.28 mg/ml for both cell lines); **E.** The cytotoxicity of PC3-TxR with Dtx alone vs Dtx+TW at concentrations of 6.25, 12, and 25 μg/ml (IC_50_ 19.70 vs 8.29, 4.08, and 2.88 nM respectively); **F.** The cytotoxicity of Du145-TxR by Dtx alone compared to its combination with TW at concentration of (12.5, 25 and 50 μg/ml) (IC_50_ >100 and 23.52 ± 1.66, 5.76 ±1.34 and 2.20 ± 1.22 with combination respectively).

The effects of Dtx on cell viability in sensitive and resistant cells (PC3 and PC3-TxR; Du145 and Du145-TxR cells) are shown in Figures 1A and 1B. The IC_50_ of Dtx or IC_50D_ was 8 and > 40 fold higher in PC3-TxR and DU145-TxR than their corresponding sensitive cell lines (PC3 and DU145), respectively. The cytotoxicity of TW were also tested which showed consistently high IC_50_ in all four cell lines (PC3/PC3-TxR and DU145/DU145-TxR; Figures 1C and 1D).

The combination of TW + Dtx was able to remarkably reverse the resistance of both resistant cell lines to Dtx (to about the same level as the sensitive cell lines) with CE of 8.17 and >38 fold for PC3-TxR and DU145-TxR cells respectively (Figure 1E and 1F). Such chemosensitizing effects were accomplished at TW concentration of 12.5 μg/ml (<50% of its own direct cytotoxicity, since IC_50_ of TW was 48.3 or 277 μg/ml for PC3-TxR and DU145-TxR, respectively).

The absorbable components (from permeate of the Caco-2 cells) in combination with Dtx was found to yield a CE = 6.8 fold in comparison to Dtx alone, indicating that the major active components could be orally absorbable (see Supplemental Figure S2).

### P-gp inhibition and Dtx accumulation

**Figure 2:**
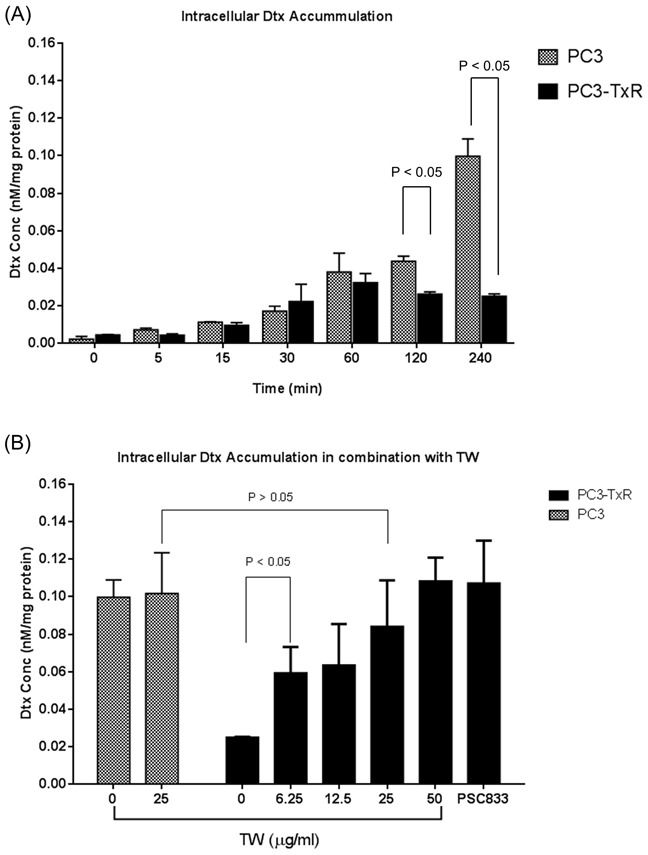
Intracellular accumulation of docetaxel (Dtx, after incubating with 100 nM Dtx) in PC3 and PC3-TxR cells **A.** Intracellular Dtx concentration in various time points showing significant higher Dtx concentration in PC3 cells; **B.** Intracellular Dtx concentration after incubating Dtx + TW (0-50 μg/ml) for 240 min. TW increased Dtx concentration in a dose dependent manner. Data expressed as mean + SD (n = 3).

The intracellular Dtx concentration in PC3 cell was found to be increased in a time dependent manner up to 240 min, while its concentration was stable after 2 hours incubation in PC3-TxR cell (see Figure 2A). Comparatively at 240 min, the concentration of Dtx in the PC3 cells was significantly higher than that in PC3-TxR cell (Figure 2A). Since Dtx is a substrate of P-gp which is overexpressed in PC3-TxR cells (see Supplementary Figure S1), its lower intracellular Dtx concentration is most likely a result of P-gp induction. However, such concentration was significantly changed when TW was added. The intracellular Dtx concentration increased in a dose dependent manner when combined with TW. Also at a TW concentration of 50 μg/ml, the intracellular Dtx concentration was increased to a similar level as that in the positive control (2.5 μg/ml of PSC833; Figure 2B). Such an increase most likely was due to inhibition of P-gp efflux by TW.

**Figure 3:**
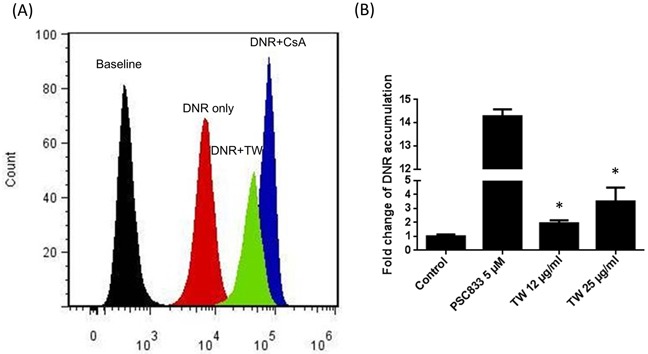
Inhibition of P-gp by TW **A.** Flow cytometric histograms of intracellular doxorubicin fluorescence for K562/Dox cells in suspension culture showing that cyclosporine (a P-gp inhibitor) and TW increased the doxorubicin accumulation; **B.** The concentration-effect TW or PSC833 on P-gp substrate doxorubicin accumulation in K562/Dox cells. PSC833 inhibited Dox efflux and lead to high intracellular concentration. TW also significantly inhibited the P-gp activity, although the potency is lower than PSC833.

Figure 3 further shows the inhibition of DNR efflux in K562/Dox (P-gp overexpressed cells) by PSC833 and TW (data are expressed as the percent inhibition of the positive control of PSC833, a known P-gp inhibitor). When incubated with TW at concentrations of 12 - 25 μg/ml, the accumulation of intracellular DNR concentration was 1.9 and 3.5 times higher than that of control (P < 0.05, 1-way ANOVA) indicating its suppression or inhibition of P-gp activity.

### Microarray analysis and new mechanisms

**Figure 4:**
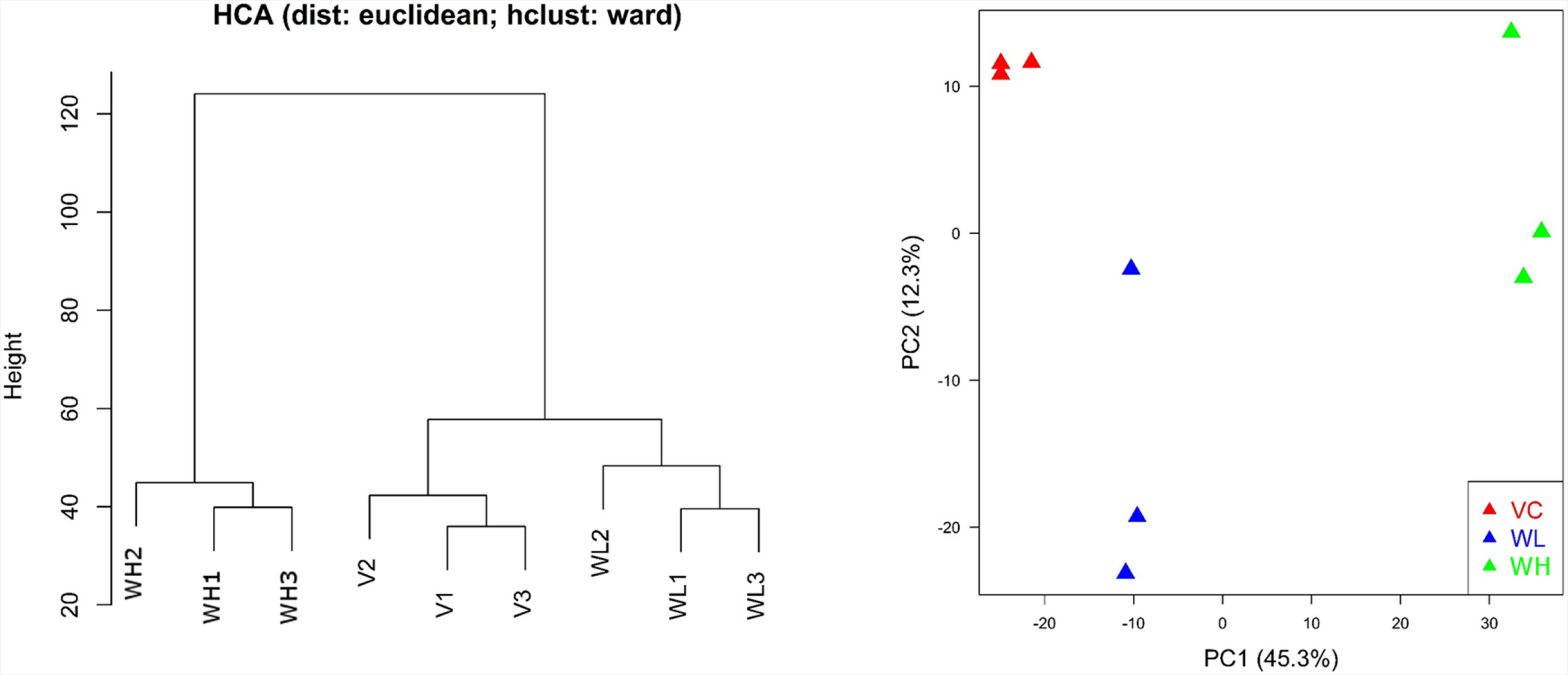
Hierarchical clustering analysis (HCA) and principal component analysis (PCA) for gene expression profiles in PC3-TxR treated with TW (VC: vehicle group; WL: TW low dose group, 0.1 mg/ml; WH: TW high dose group, 1 mg/ml) PC1 and PC2 represent first principal component and second principal component respectively. Samples for the same treatment groups were clustered together indicating reliable data quality. Different groups clearly separated from each other indicating a strong treatment effect for TW.

Hierarchical clustering analysis (HCA) and principal component analysis (PCA) showed that the quality of microarray data generated in this study appeared reliable. Visual inspection of the PCA plot yielded a straightforward diagnosis of the sources of variance in these datasets. The PCA was based on log2 intensity as shown in Figure 4. Samples for the same treatment group were clustered together indicating good reproducibility of microarray experiments.

The number of differentially expressed genes varied dramatically in a dose dependent manner. The high dose TW treatment resulted in much higher numbers of genes differentially expressed than that of low-dose TW group (1,168 genes corresponding to 1,912 probe-sets versus 158 genes corresponding to 206 probe-sets for high and low dose groups respectively; Table 1).

**Table 1:** Number of differentially expressed genes of each treatment group

Treatment	No. of probe-sets (*p*<0.05, |FC|>1.5)	No. of genes (*p*<0.05, |FC|>1.5)
VC	-	-
WL	206	158
WH	1912	1168

* VC: control group; WL: TW 100 μg/ml; WH: TW 1000 μg/ml

**Figure 5:**
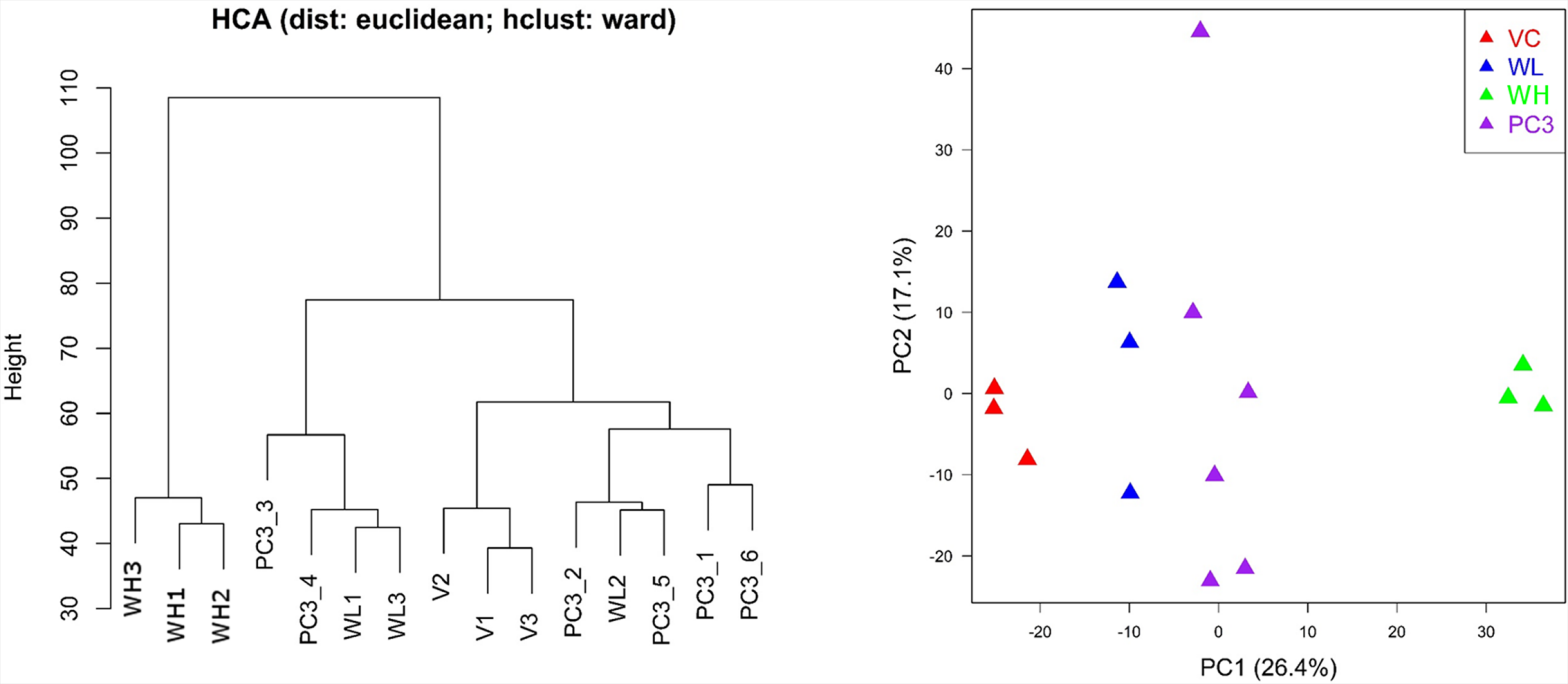
Hierarchical clustering analysis (HCA) and principal component analysis (PCA) for gene expression profiles in PC3 and PC3-TxR cells (VC: vehicle group; WL: TW low dose group, 0.1 mg/ml; WH: TW high dose group, 1 mg/ml) PC1 and PC2 represent first principal component and second principal component respectively. The distance of expression profile in PC3-TxR became closer to PC3 cells (the sensitive cells) after treated by TW.

PCA results showed that the expression profiles are remarkably different between PC3 and PC3-TxR cells. The expression profile of PC3-TxR cells in the low dose TW group was more similar to PC3 cells (Figure 5).

Two KEGG pathways were significantly enriched (P < 0.05) from the low dose TW treatment, while 16 KEGG pathways were significantly enriched with differentially expressed genes from the high dose TW treatment (Table 2). Table 2 lists the two KEGG pathways and top five KEGG pathways (significantly enriched with the differentially expressed genes) from the low dose and high dose groups respectively. Among these pathways, cancer pathways were significantly affected by both doses of TW treatments.

**Table 2:** Top 5 KEGG pathways enriched with differentially expressed genes and their corresponding Fisher's exact test p values

Treatment	KEGG pathway name	*p*-value	The number of DEGs found in the KEGG pathway	Ratio (%)
WL	Pathways in cancer	5.5e-4	11	6.8
	Cytokine-cytokine receptor interaction	2.1e-3	9	5.8
	Pathways in cancer	1.0e-12	60	4.7
	Small cell lung cancer	5.3e-7	21	1.7
WH	p53 signaling pathway	1.8e-6	18	1.4
	TGF-beta signaling pathway	2.1e-4	17	1.3
	Cell cycle	2.6e-4	21	1.7

The ratio is calculated by dividing the number of differentially expressed genes by the total number of genes involved in the pathway.

* VC: control group; WL: TW 100 μg/ml; WH: TW 1000 μg/ml

The top five KEGG pathways enriched with the dose dependent DEGs are shown in Table 3. The pathways relating to cancer were most significantly enriched, in which a total of 51 genes were enriched. Most of these were related to regulation of apoptosis, expression of drug transporters and cell cycling.

**Table 3:** Top 5 KEGG pathways enriched with genes showing dose-responsive changes

KEGG pathway	*p* value	Number of DEGs found in the KEGG pathway	Ratio (%)
Pathways in cancer	2.1e-4	9	10.1
Cytokine-cytokine receptor interaction	2.1e-3	7	7.9
Basal cell carcinoma	2.9e-3	4	4.5
Retinol metabolism	3.3e-2	3	3.4
Hedgehog signaling pathway	3.5e-2	3	3.4

The ratio is calculated by dividing the number of differentially expressed genes by the total number of genes involved in the pathway.

From these DEGs, a total of 10 genes were found to be potentially linked with chemoresistance and their expression profiles showed dose dependent manner (see Supplementary Figure S3, Table 4). The expression level of these 10 genes was verified by using real time PCR. The direction (up or down) of regulation is consistent between microarray and real time PCR analysis. Although the fold changes of the genes were different, a good correlation was identified between microarray and real time PCR (see Supplementary Figure S4, r^2^=0.583, P < 0.001, Pearson correlation).

**Table 4:** Summary of rtPCR results of ten dose dependent DEGs

Gene	Real time PCR (fold change)				
	PC3*	PC3-TxR treated with TW (μg/ml) for 6 h			
		0	6.25	12.5	25
Homo sapiens DNA-damage-inducible transcript 4 (DDIT4)	1.99 ± 0.51	1.00 ± 0.06	1.25 ± 0.09	1.58 ± 0.48	1.17 ± 0.17
Homo sapiens epithelial-specific transcription factor ESE-1a (ESE-1)	14.84 ± 1.17	1.01 ± 0.13	1.66 ± 0.05	2.12 ± 0.91	2.43 ± 0.75
Homo sapiens prostate differentiation factor mRNA (HPDF)	9.05 ± 0.06	1.01 ± 0.18	1.35 ± 0.10	1.82 ± 0.74	1.58 ± 0.40
Homo sapiens heme oxygenase 1 (HO-1)	1.68 ± 0.82	1.07 ± 0.44	0.71 ± 0.31	0.69 ± 0.27	0.52 ± 0.25
SLC16A6	0.30 ± 0.21	1.02 ± 0.23	0.93 ± 0.18	0.96 ± 0.11	0.52 ± 0.04
Homo sapiens hedgehog-interacting protein mRNA (HHIP)	0.13 ± 0.00	1.00 ± 0.10	0.94 ± 0.08	1.02 ± 0.42	0.63 ± 0.14
Homo sapiens TXK tyrosine kinase (TXK)	0.43 ± 0.02	1.02 ± 0.21	1.03 ± 0.04	0.78 ± 0.29	0.76 ± 0.37
Human connective tissue growth factor (CTGF)	0.45 ± 0.02	1.01 ± 0.16	0.61 ± 0.02	0.35 ± 0.02	0.29 ± 0.10
Homo sapiens cyclin E2 splice variant 1 mRNA (CCNE2)	2.61 ± 0.81	1.02 ± 0.26	0.94 ± 0.01	0.54 ± 0.09	0.43 ± 0.23
Homo sapiens inhibitor of DNA binding 3, dominant (ID3)	1.29 ± 0.08	1.01 ± 0.05	0.78 ± 0.05	0.89 ± 0.29	0.32 ± 0.15

* Compare to PC3-TxR cells without any treatment. The data are expressed as Mean ± SE (n = 3).

### Real time PCR

The expression of the ten genes from PC3-TxR and PC3 cells treated by TW at concentrations of (6.25, 12.5, and 25 μg/ml) are shown in Table 4. Among these genes, expressions of HO-1 and SLC16A6 were different from that in microarray study, i.e., they were down-regulated rather than up-regulated. The level of DDIT4, ESE-1, and HPDF were higher in PC3 than PC3-TxR cells. After treatment by TW, these three genes were up-regulated. The expression of SLC16A6, HHIP, TXK and CTGF were lower in PC3 cells than PC3-TxR cells, and they were down-regulated by the treatment of TW. HO-1, CCNE2 and ID3 were also suppressed by TW. However their expressions were higher in PC3 than PC3-TxR cells.

### Efficacy study and quality control of TW extract

CE was used as a biological assay for quality control of the TW extract. It was determined at various time points such as during the in vitro studies, prior to MTD and efficacy studies. The average CE was 8.22 with CV (coefficient of variation) of 23% indicating a consistent activity throughout different times.

Both the single and multiple dose studies showed MTD to be around 500 mg/kg, which was used for the subsequent xenograft study. The subsequent in vivo efficacy study further verified this MTD as body weight loss of the mice was less than 10% when received 500 mg/kg TW for two weeks (See Supplementary Figure S5).

**Figure 6:**
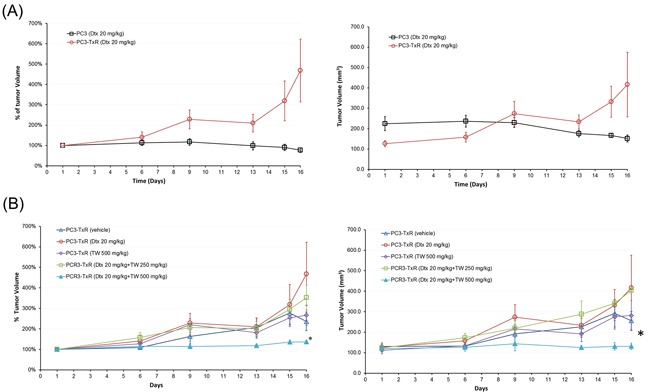
Effect of Dtx, TW, or their combination in SCID mice implanted with PC3 or PC3-TxR cells **A.** Percentage (left) and absolute (right) tumor volume over time in SCID mice implanted with PC3 and PC3-TxR cells treated with i.v. 20 mg/kg Dtx alone after 3 doses; **B.** Percentage (left) and absolute (right) tumor volume changes in SCID mice implanted with PC3-TxR cells treated with saline, Dtx alone (20 mg/kg, i.v. every week), TW alone (500 mg/kg p.o. daily), Dtx (20 mg/kg, i.v. weekly + TW (250 mg/kg p.o. daily) and Dtx (20 mg/kg i.v. weekly) + TW (500 mg/kg p.o. daily). The drug combination significantly retarded the tumor growth. *AUC of tumor volume under the treatment curve from optimum dose (Dtx + TW 500 mg/kg) group was significantly less than that of other groups (P<0.05, 1-way ANOVA).

PC3-TxR Dtx was found to significantly inhibit the PC3 implanted cell tumor growth (the sensitive cells), but not the PC3-TxR cells (Figure 6A). Thus our PC3-TxR xenograft model for testing the treatment effect in resistant tumor was valid. Using such a model, our results showed that treatment with Dtx or TW alone or TW at low dose (250 mg/kg) + Dtx failed to suppress the tumor size after 15 days of dosing. However, the combination of an optimum dose, i.e., high TW (500 mg/kg) + Dtx treatment significantly retarded the PC3-TxR cell tumor growth (P < 0.05 for area under the curve in comparison with that from other treatment groups, Figure 6B).

## DISCUSSION

This is the first demonstration of strong CE from TW when combined with Dtx in prostate cancer cells/tumor resistant to Dtx. Based on our P-gp assay work and microarray/quantitative PCR analysis, multi-mechanisms have been found to be associated with the chemosensitizing effect. The multi-mechanistic effect of TW may have important therapeutic implication since single targeted approaches (e.g., pure P-gp inhibition) have not been proven to control cancer.

The cytotoxicity study showed both PC3-TxR and DU145-TxR cells were resistant to docetaxel (much higher IC_50D_ were observed for the resistant cell lines using the cytotoxicity assay). TW successfully reversed such resistance indicating the potential chemosensitizing effect of TW. However, TW alone has similar cytotoxicity, especially for DU145 and DU145-TxR cell lines.

Quality control is very important for herbal medicine and quantification of chemical marker compounds is commonly used. However, this approach is practically not feasible for TW, since over 300 compounds have been identified [21]. Thus the use of CE as a biological assay was utilized for the quality measurement of our TW extract (in conformance to “FDA Guidance on Botanical Drug Development”, FDA, 2015). Similar CE values (CV<30%) have been observed for our TW extract when tested at different times, indicating our TW extract method can yield a relatively consistent and stable value throughout the study period.

In our study, the suppression of P-gp activity by TW is supported by the increase in intracellular concentration of Dtx in PC3-TxR cells (the concentration of Dtx was remarkably increased to a similar level as that in the PC3 cells). Such effect is further substantiated by the studies in K562/Dox cells (a P-gp overexpressed cell line) [24].

In addition to suppression of P-gp, many other mechanisms may be involved in chemoresistance [25]. Since TW contains more than 300 components, some are likely to target different mechanisms of resistance [20]. Our microarray data and real time PCR results support multiple targets by TW. The PCA results from the microarray data also showed that the distance of expression profile in PC3-TxR when treated with TW became closer to PC3 cells (the sensitive cells), consistent with TW's chemosensitizing action in PC3-TxR cells.

Detail pathway analysis showed that certain pathway relating to anticancer effect are enriched by the treatment of TW, although the exact pathways involved in the chemoresistance have not been identified by the present study. Nevertheless, TW has been found to affect certain genes relating to drug resistance of cancer chemotherapy. These specific genes (i.e., dose dependent DEGs) include SLC16A6, CTGF, ESE-1a, CCNE2 and HO-1 and their pertinent information relevant to chemoresistance and CE are summarized below.

SLC16A6, a monocaroboxylate transporter, the expression of which has been previously found to be significantly increased in paclitaxel-resistant variant of W1 ovarian cancer cell line [26]. In our study, the SLC16A6 expression in the PC3-TxR cells was found to be 3 times higher than the sensitive PC3 cells. After treatment with TW, the level was decreased to that of the sensitive PC3 cells.

Connective tissue growth factor (CTGF) is a secreted protein modulating the tumor microenvironment and may be involved in the conversion of cancer cells to cancer stem cells which is a known mechanism of chemoresistance [27, 28]. Overexpression of CTGF can increase the resistance to paclitaxel-induced cell apoptosis. The inhibition of CTGF sensitized the activity of gemcitabine in pancreatic ductal adenocarcinoma [28–31]. In our study, TW suppressed the expression of CTGF in a dose dependent manner.

Epithelial-specific transcription factor, ESE-1a, is an E26 transformation-specific (Ets)-related transcription factor, which can induce endogenous TGFβ type II receptor expression and restore the TGFβ signaling pathway [32]. TGFβ is a tumor repressor and its inhibition is known to be related to cancer progression. In our study, ESE-1a was significantly lower in PC3-TxR compared to PC3 cells. After treatment with TW, ESE-1a expression was restored in a dose dependent manner.

Cyclin E2 (CCNE2) is involved in the G1/S portion of the cell cycle and the over expression of CCNE2 could lead to resistance in prostate and breast cancer cells [33, 34]. Prostate cancer is an endocrine responsive tumor and may become resistant to endocrine therapy when it becomes metastatic castration-resistant prostate cancer with over expression of CCNE2 [35]. In our present study, the expression of CCNE2 decreased by TW treatment which may increase the sensitivity to hormone treatment of prostate cancer.

Heme oxygenase-1 (HO-1) possesses antioxidative and antiapototic activity and may also contribute to the development of acquired chemoresistance in solid tumor tissue as well as acute myeloid leukemia cells [36, 37]. HO-1 has been found in the adhesive and morphological properties of prostate cancer cells and can be a potential molecular target to restore the chemosensitivity in prostate cancer [38]. HO-1 induction has been found to increase E-cadherin and β-catenin levels leading to a striking remodeling of E-cadherin/β-catenin based cell adherens junctions and a markedly change in cell morphology [39]. In our study, TW was found to decrease the expression of HO-1 and might be able to reverse the chemoresistance in prostate cancer cells.

In addition to the above genes, several genes relating to the cell growth were also down-regulated by TW. These include DDIT4, HPDF, HHIP, ID3 and TXK tyrosine kinase. There genes may also be potentially involved in the chemoresistance of PC3-TxR cells [40–42].

The above information on the effect of TW on these new candidate genes provides an understanding of their potential roles in prostate cancer progress and drug resistance, although further verification is needed. Nevertheless, based on a number of the dose-dependent changes observed, TW is likely to be capable of targeting these genes.

The *in vivo* efficacy study showed significant tumor growth retardation by the treatment of TW (high dose) + Dtx combination, compared to other treatments. Since the combination of Dtx with TW (low dose) group failed to retard tumor growth, a sufficient dose of TW would be essential for successful tumor inhibition. This relatively high dose (500 mg/kg) obviously cannot be translated to human use. As the TW extract contains many inactive substances, a further refinement of the extract should be able to reduce the “inactive” components and reduce the dosage for human use in future.

The last data time point of the tumor growth curve from vehicle treatment showed a drop in tumor size (however without statistical significant difference). This drop probably reflected an experimental error in the measurement of the tumor size at that point. However, our analysis of the treatment effect was based on measurements over 6 time points rather than one time point (e.g., the last time point). Thus the vehicle group did not affect our finding of the significant treatment effect from the combination of TW + Dtx.

Our positive *in vitro* and *in vivo* results as well as potential advantage of multi-mechanisms from TW in combination with Dtx indicate that TW may be considered as a promising herbal product for use in combination with Dtx for resistant prostate cancer therapy. Since TW has already been marketed for decades in China for the treatment of rheumatoid arthritis, chronic nephritis, and several skin disorders, its safety and toxicity in human subjects have been already tested and accepted (although there was a report of potent inhibitory effect on the clonogenic response of human bone marrow cells when assessed *in vitro* [43]. Such wide human usage experience together with the present results can pave the way to future clinical trial of TW for prostate cancer resistant to Dtx, which has no effective treatment so far.

Our study showed that *Tripterygium wilfordii*, a tradition Chinese medicine, appears to be capable of sensitizing the chemotherapeutic effect of docetaxel in resistant prostate cancer cells via multiple mechanisms. While our present study has only focused on resistant prostate cancer cells, future similar work with other herbs and other resistant cancer cells may lead to discovery of other natural products to overcome chemoresistance, a major problem facing cancer chemotherapy today.

## MATERIALS AND METHODS

### Materials

The debarked roots of TW raw herb was purchased from Jilin, China (Sanyuan Pharmac. Co. Ltd., Jilin, China) and authenticated by Prof. Zhao Zhongzhen of Baptist University of Hong Kong (Hong Kong, China). Dtx was purchased from Sigma-Aldrich Co. (St Louis, MO, USA). The human androgen-independent prostate cancer cell lines DU145 and PC-3, and the human colorectal adenocarcinoma Caco-2 cell line were obtained from American Type Culture Collection (ATCC, Manassas, VA, USA). Cell culture medium RPMI-1640, DMEM, fetal bovine serum (FBS), pyruvic acid, none-essential amino acids, penicillin-streptomycin, and 0.25% trypsin-EDTA solution were purchased from Invitrogen Corp. (Carlsbad, CA, USA). The Dtx resistant cell lines (PC3-TxR and DU145-TxR) which were established using a stepwise increase in exposure to Dtx were kindly provided by Department of Medicine, University of Michigan [44]. All the solvents used were of HPLC grade purity or higher (VWR, Brisbane, CA, USA).

### Methods

#### Preparation of TW and Caco-2 permeate

The debarked roots of *Tripterygium wilfordii* were grounded (~100 g) and extracted with adequate volumes of ethanol (95%, 1.8 L) three times (0.6 L each time) after overnight maceration followed by sonication for 30 min. The solvent from the filtered extract was evaporated (Buchi Rotary Evaporator R200 System, BUCHI Corp., New Castle, DE, USA). The dry residue so obtained was fractionated by adsorbing on silica gel 60 and sequentially extracted with hexane, chloroform, acetone and ethanol (95%) to obtain fractions of increasing polarity. The ethanol extract was subsequently selected for further study, since it is less toxic to the cells and is the preferred solvent for usual herbal product manufacturing.

As CM products are generally administered orally for therapeutic use due to safety and convenience, the demonstration of the *in vivo* efficacy of their orally absorbable herbal components would be highly desirable. Thus we used a well-recognized approach of using Caco-2 monolayer model to obtain the potentially absorbable components of TW (as described by us previously) [23, 45]. Briefly, Caco-2 cells were cultured for 21 days in Transwell^®^ inserts (Corning Costar Co., Corning, NY, USA). The permeability studies were performed in transport buffer containing phosphate buffer saline (PBS) (pH 7.4) with supplement of calcium (0.8 mM) and potassium chloride (0.9 mM). TW was dissolved in PBS at 0.25 mg/ml and loaded to the donor chamber that was pre-incubated with the transport buffer at 37°C for 15 min. After incubation for 2 hours, the permeate containing the absorbable components in the receiver side was collected, separated (using the HLB Cartridge; Waters Corp., Milford, MA, USA) and then concentrated for cytotoxicity and chemosensitizing studies described below.

#### Cell cytotoxicity and chemosensitizing effect (CE)

The growth inhibitory effect of TW, Dtx, or their combination on two specific cancer cells was tested using a proliferation assay with sulforhodamine B (SRB). The PC3 and DU145 cells as well as the corresponding resistant cell lines (PC3-TxR and Du145-TxR) to Dtx were maintained in the RPMI1640 medium supplemented with 10% FBS, 100 μM streptomycin and 100 units penicillin at 37°C in a 5% CO_2_ humidified atmosphere. After achieving 80% confluence, the cells were trypsinlized and seeded at a density of 3000 cells/well in 96-well cell culture plate. After incubation for 24 hours, the cells were treated with Dtx (0.01, 0.1, 0.33, 1, 3.3, 10, 33, and 100 nM), TW (3 to 1000 μg/ml), or TW + Dtx (cells were pre-treated with TW or permeate for 2 hours prior to adding Dtx) for 72 hours with three concentrations of TW (see below). Cell viability was measured using the SRB test (see Supplementary information. The control cell viability (no Dtx) was designated as 100%. Cell viability at different Dtx concentrations was plotted. The IC_50_ was calculated using an Emax sigmoid model with the aid of GraphPad Prism software (GraphPad Software, San Diego, CA, USA).

The concentrations of TW selected for herb + drug (HD) combination study were 12.5%, 25% or 50% IC_50_ of TW (IC_50H_) to minimize toxicity. The chemosensitizing effect (CE) was expressed as CE =IC_50D_/IC_50HD_, where IC_50D_ was the drug or Dtx concentration that inhibited 50% proliferation, whereas IC_50HD_ was the Dtx concentration that inhibited 50% proliferation when in combination with TW [13]. Both TW raw extract as well as the permeate through Caco-2 cells were used. To minimize day to day variation associated with cell culture studies, each comparative study was performed on the same day whenever possible.

#### Dtx accumulation assay

The effect of TW on Dtx intracellular accumulation (in PC3-TxR cells and PC3 cells) was tested. The PC3 and PC3-TxR cells were seeded in 6-well plate with density of 10^4^ cells/ml and grew for 24 hours and then treated with 100 nM Dtx. The cells were rinsed with cold PBS to wash off the attached Dtx to cell membrane and then collected at 0, 5, 15, 30, 60, 120, and 240 min. Whereas for Dtx in combination with 0, 6.25, 12.5, 25, and 50 μg/ml TW or PSC833 (2.5 μg/ml, served as positive control), they were incubated for 240 min, after which the cells were washed and lysed. The protein level was measured using a bicinchoninic acid (BCA) test (Bio Rad, Hercules, CA, USA). The intracellular concentration of Dtx was determined using an HPLC-MS/MS method. The intracellular concentration was normalized per protein concentration (see Supplementary method).

#### Daunorubicin accumulation assay

P-glycoprotein (P-gp), also known as multidrug resistance protein 1 (MDR1) or ATP-binding cassette sub-family B member 1 (ABCB1), is a well-known efflux membrane transporter, the overexpression of which was found to be a main mechanism of chemoresistance [3, 24]. The expression of P-gp in PC3-TxR cells was found to be much higher than that in PC3 cells (see supplementary Figure S1) indicating the resistance of PC3-TxR to Dtx is at least partially modulated by P-gp.

To verify the inhibitory effect on P-gp by TW, a P-gp over expressing K562/Dox leukemia cell line was used. The ability of TW to inhibit the P-gp mediated drug efflux was assessed by a flow cytometry based drug accumulation assay. Daunorubicin (DNR) was used as the fluorescent marker and PSC833 used as the positive control. The K562/Dox cells were maintained in RMPI 1640 with 10% FBS at 37°C. Initially the cells were collected by centrifuging at 1,000 rpm for 5 min at room temperature and re-suspended using RMPI 1640 medium to adjust the cell density of 5×10^3^ cells/ml. Then the cells were incubated with DNR (5 μM) alone or in the presence of various concentrations of TW for 2 hours. They were then collected by centrifugation and re-suspended using cold PBS. The intra-cellular fluorescent intensity was measured using a Becton Dickinson FACS Calibur flow cytometer (BD Biosciences, San Jose, CA, USA) equipped with an Ultra Violet Argon laser (excitation at 488 nm, emission at 585/542 nm). The Log fluorescence intensities of those individual cells treated with TW were recorded and normalized as percent of the positive control.

#### Identification of other molecular mechanisms by microarray and real time PCR

In order to reduce the possibility of false negative results and identify the most significant DEGs, a concentration dependent design using TW concentrations at 100 and 1000 μg/ml were used in the microarray study. Subsequently, the results were confirmed with real time PCR study. In addition, TW at lower concentrations (6.25, 12.5 and 25 μg/ml, as per previous cytotoxicity study) were tested using real time PCR approach.

##### Microarray study

***RNA isolation and gene expression profiling*** Since many mechanisms can contribute to resistance, a microarray process/analysis procedure was utilized to identify the potential genomic mechanisms related to the chemosensitizing effect of TW in PC3-TxR cells by determining the gene expression profile [23]. Briefly, PC3-TxR cells were seeded at a density of 1×10^5^ cells/mL and cultured for 24 h. The cells were then treated with TW at concentrations of 100 and 1000 μg/ml (initially dissolved in DMSO and diluted to the desired concentration using the medium) for 6 hours. DMSO (0.5%) was used as the negative control (n = 3). Total RNA was extracted using RNeasy Mini Kit (QIAGEN, Valencia, CA, USA) according to the manufacturer's protocol. The RNA was measured and the quality was checked using the RNA 6000 LabChip and Agilent 2100 BioAnalyzer. Only the high-quality RNA samples (e.g. RNA integrity number greater than 9.0) were used for subsequent microarray procedure (performed at the Functional Genomics Core, Beckman Research Institute, City of Hope Comprehensive Cancer Center, Duarte, CA, USA). Affymetrix Human Genome U133 Plus 2.0 arrays (Affymetrix, Santa Clara, CA, USA) containing 54,675 probe sets detecting over 47,000 transcripts were used. The RNA samples were randomized and blinded prior to the microarray processing/analysis. The assay was performed using an Affymetrix GeneChipH 3000 7G scanner (see Supplementary method).

***Microarray data processing and quality assessment*** Statistical testing and additional analysis of the microarray data were conducted using the R package (Lucent Technologies, Murray Hill, NJ, USA). The probe-set level expression data were summarized with Robust Multichip Average (RMA) by taking all 9 microarrays together. The log2-transformed expression intensities of 54,675 probe sets from the nine microarrays were used to calculate the Pearson Correlation Coefficient and Ward's Minimum Variance method was used to calculate the distance between samples. Principal component analysis and hierarchical clustering analysis combined with heatmap was used to assess the quality of the microarray data (by evaluating the reproducibility and variation of three replicates within each group and the differences among the three groups: control, 100 μg/ml and 1000 μg/ml TW groups).

In addition to the microarray data obtained from the PC3-TxR cells, gene expression profiles of PC3 cells from six microarrays were included (3 arrays from GSE41445 and 3 arrays from GSE33455, Gene Expression Omnibus). The PC3 microarray data were utilized for principle component analysis with results obtained from PC3-TxR cells after per gene per data set mean-zero normalization.

***Functional analysis of differentially expressed genes with DAVID*** The differentially expressed genes (DEGs) between the treatment and control group were identified using cutoffs of t-test p < 0.05 and fold change > 1.5. The DEGs and probe sets were selected separately by comparing each treatment group with the control group. The expression profiles of the DEGs (probe sets) were imported to the DAVID website, a web-based functional annotation tool (http://david.abcc.ncifcrf.gov/), to identify pathways significantly enriched with the DEGs. (KEGG, Kyoto Encyclopedia of Genes and Genomes, is a powerful database for understanding and simulating functional behaviors of cells or organisms from their genome information). The lists of differentially expressed genes were also input to the Kyoto Encyclopedia of Genes and Genomes (KEGG) database. The probe sets in each treatment group were mapped to the HUGO gene symbols within the KEGG database, and the pathways enriched with the differentially expressed genes according to the Fisher's exact test p values were considered as effect from treatment. When multiple probe-sets were mapped to the same gene symbol, only the probe-set with the maximal absolute log2 fold change was kept for identifying enriched KEGG pathways. The probe-sets that did not map to any gene symbol were discarded. The pathways were listed according to their corresponding p-values.

***Identification of dose responsive genes and pathways most significantly enriched*** Genes or pathways with dose-dependent changes are more likely to be associated with a valid pharmacological effect of drug treatment. The list of DEGs from TW low (WL) and high dose (WH) treatment were determined by comparing with the control (pairwise comparison). To identify the mechanism of chemosensitizing effect by TW, the dose dependent differentially expressed genes were identified by overlapping the two lists of DEGs. The dose dependent DEGs were then mapped to the KEGG database.

##### Real time-PCR study

***Verification of Microarray DEG Expression by Real-Time PCR*** The dose dependent DEGs were further evaluated according to their potential function associated with chemoresistance. Those genes so identified from microarray study were selected and validated using real time PCR with a SYBR green protocol (Applied Biosystems 7300 Real-Time PCR System, Applied Biosystems, Carlsbad, CA, USA). The PC3-TxR cells were treated with TW at concentrations of 100 and 1000 μg/ml for six hours. In addition, PC3-TxR cells were treated with other three lower concentrations (6.25, 12.5, and 25 μg/ml as per our chemosensitizing effect studies). The mRNA from PC3-TxR cells as well as non-treated PC3 cells was then extracted and followed by reverse transcription. The gene expression was detected using real time PCR. The data for each sample were displayed as a melting curve and the “crossing point”, representing that the RNA expression level was determined. Then, the expression fold changes of the DEGs were quantified with the GAPDH as the reference (normalizing control). The results were further adjusted in comparison to that from the negative control.

The expression profiles of these genes in PC3-TxR cells were also compared to their expression levels in PC3 cells.

#### Efficacy study *in vivo*

Following the *in vitro* CE and mechanistic studies, the *in vivo* efficacy studies of TW and its combination with Dtx were performed in severe combined immunodeficiency (SCID) mice. Prior to the efficacy study, the maximum tolerated dose of TW was determined using the CD-1 mice. All animal studies were approved by the Institutional Animal Care and Use Committee (IACUC) at Western University of Health Sciences (Pomona, CA, USA) with all procedures complied with the study guidelines of IACUC. The quality of the TW extract was also examined by determination of its CE value prior to the animal studies.

##### Determination of maximum tolerated dose

Since the oral tolerance and effective dose of TW *in vivo* were unknown, the oral maximum tolerated dose (MTD) in mice was first determined using escalating single oral doses of 100, 250, 500, 750 and 1000 mg/kg and multiple doses of 62.5, 125, 250 or 500 mg/kg daily for 7 days in 42 male CD-1 mice (Charles River Laboratories International Inc., Wilmington, MA, USA). The TW extract prepared (see section 2.2.1) was first dissolved in ethanol to make a stock solution of 500 mg/ml and then diluted with saline to final concentration of 250 mg/ml before dosing. The MTD was defined as the dose that led to no death, no more than 10% or greater retardation of body weight gain as compared to control animals and no overt organ dysfunction or side effects.

##### Xenograft study

Male SCID mice of 15–20 g at 4–6 weeks of age (Taconic Farms Inc., Oxnard, CA, USA) were housed in cages with HEPA-filtered air (12-hr light/dark cycle). The PC3 and PC3-TxR cells were cultured as before and then harvested and suspended in Matrigel (BD Biosciences, San Jose, CA, USA) and PBS (1:1). Approximately 1×10^6^ cells were then subcutaneously implanted into the flanks of mice. When the xenograft tumors reached ~120 mm^3^ (calculated using the formula for a semi ellipsoid: Volume = Width^2^ × (Length/2)), the mice were randomized to receive six treatments: (1) PC3-TxR, control (vehicle group, i.v. saline and p.o. 50% ethanol daily); (2) PC3, Dtx (20 mg/kg, i.v., once per week); (3) PC3-TxR, Dtx (20 mg/kg, i.v., once per week); (4) PC3-TxR, TW (500 mg/kg, p.o., daily); (5) PC3-TxR, Dtx (20 mg/kg, i.v., once per week) + TW low dose (250 mg/kg, p.o., daily); and (6) PC3-TxR, Dtx(20 mg/kg, i.v., once per week) + TW high dose (500 mg/kg, p.o., daily). For groups 5 and 6, the dose of Dtx was 20 mg/kg (i.v., once per week), while the doses of TW were 250 mg/kg and 500 mg/kg (p.o., once daily) for low and high dose groups respectively.

### Statistics

All data from the study are expressed as mean ± standard error (SE). The results among different groups were compared using one-way analysis of variance (ANOVA), followed by the post-hoc Bonferroni test for multiple comparisons. The difference between two independent groups was compared by the Student t test. The consistency between the microarray and real time PCR was compared by Pearson correlation. The areas under the curve of tumor growth versus time from different treatment groups were calculated using linear trapezoidal method and compared among these groups using one way ANOVA with post hoc multiple comparisons using Bonferroni test. A probability value (P) < 0.05 was considered statistically significant for all tests. All analysis was performed with SPSS software (version 12.0; SPSS, Chicago, Illinois, USA).

## SUPPLEMENTARY MATERIALS METHODS AND FIGURES


