# Supplementary material for: Overcoming chemoresistance in prostate cancer with Chinese medicine Tripterygium wilfordii via multiple mechanisms
Source: Oncotarget. 2016 Jul 28;7(38):61246–61. doi: 10.18632/oncotarget.10868 (PMC5308648; doi:10.18632/oncotarget.10868)
Supplement: Supplementary file 1 [file oncotarget-07-61246-s001.pdf]

Overcoming chemoresistance in prostate cancer with Chinese medicine Tripterygium wilfordii via multiple mechanisms

SUPPLEMENTARY METHODS

SRB assay

Cells were fixed with 10% TCA for 1 hour at 4°C and washed with water. The residue in the well was stained with SRB solution 0.4% (w/v) in 1% acetic acid for 0.5 h. Then the SRB was re-dissolved in 10 mM of Tris buffer (pH 8.0) after removing the supernatant. The optical density at 590 nm was determined using a 96-well plate reader.

HPLC-MS/MS

To 0.1 ml of cell lysate, 0.3 ml of tertiary butyl methyl ether was added and votexed for 3 min. The upper organic layer was separated and evaporated to dryness. The residue was reconstituted using 0.1 ml of 80% acetonitrile and 10 μl was injected to an HPLC-MS/MS system for quantification. Paclitaxel was used as internal standard. The typical MS conditions of paclitaxel and docetaxel are as follows (the precursor ions are $[M+Na]^+$ for both compounds).

Microarray

The cRNA synthesis and labeling were carried out following the Affymetrix GeneChip 3' IVT Express standard preparation protocol using 200 μg of total RNA from each sample, along with polyA spike-in controls. The cRNA samples were converted to double-stranded cDNA. After second-strand synthesis, the cDNA was purified with the GeneChip sample cleanup module (Affymetrix). Biotinylated cRNAs were then synthesized by in vitro transcription. For each sample, 10 mg of biotinylated cRNA along with hybridization spiked controls (bioB, bioC, bioD, and cre) was hybridized with Affymetrix Human Genome U133 Plus 2.0 array for 16 h at 45°C. Following hybridization, arrays were washed, stained, and then scanned with an Affymetrix GeneChipH 3000 7G scanner.

Compounds	Precursor ion (m/z)	Product ion (m/z)	DP (V)	EP (V)	CE (V)	CEP (V)	CXP (V)
Paclitaxel	876.067	308.100	86	7.5	39	34	6
Docetaxel	830.090	304.200	76	8.0	33	34	6

DP, declustering potential; EP, entrance potential; CEP, collision cell entrance potential; CE, collision energy; CXP, collision cell exit potential.

SUPPLEMENTARY FIGURES

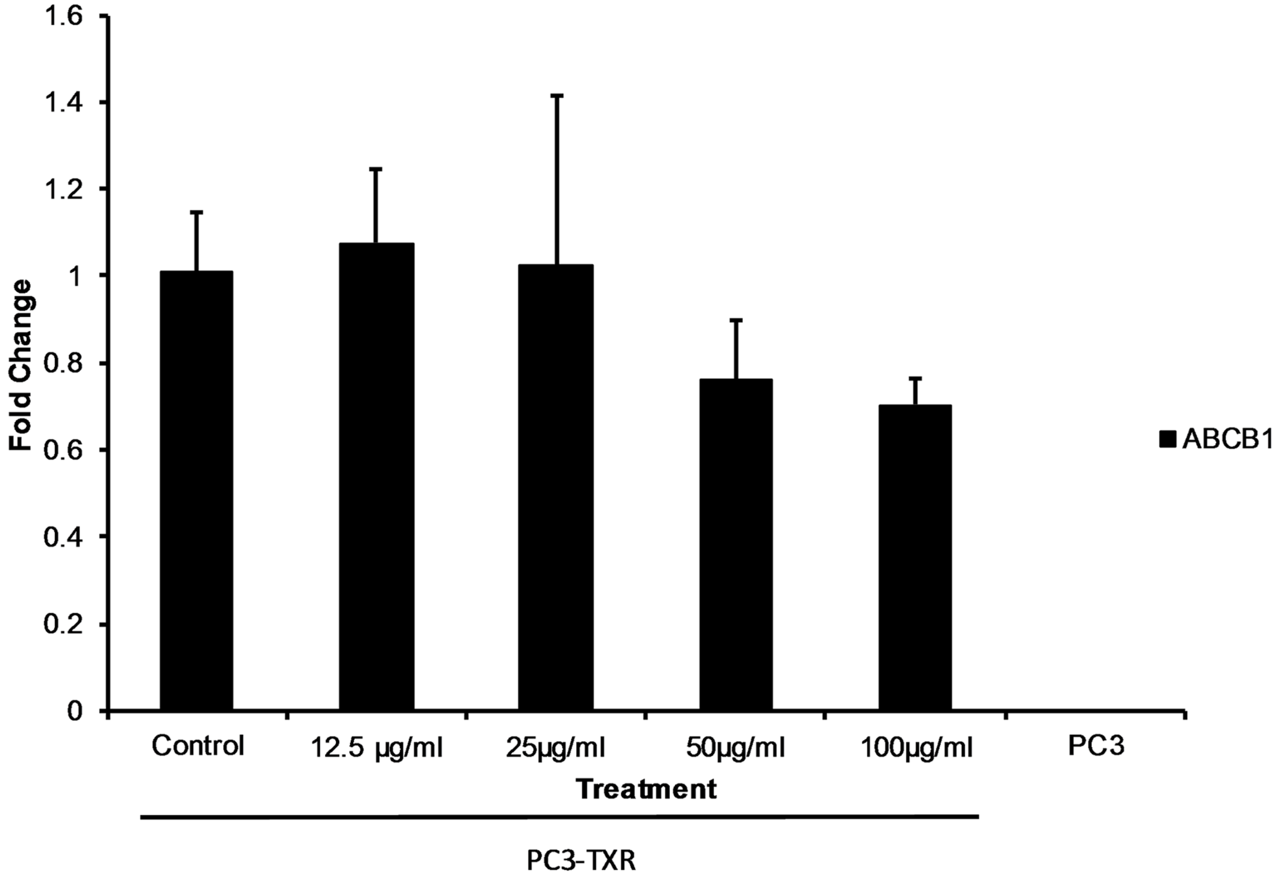

Supplementary Figure S1: Real time PCR results of P-gp expression level in PC3 and PC3-TxR cells. P-gp is overexpressed in PC3-TxR cells and TW has no effect on its expression at low concentrations.

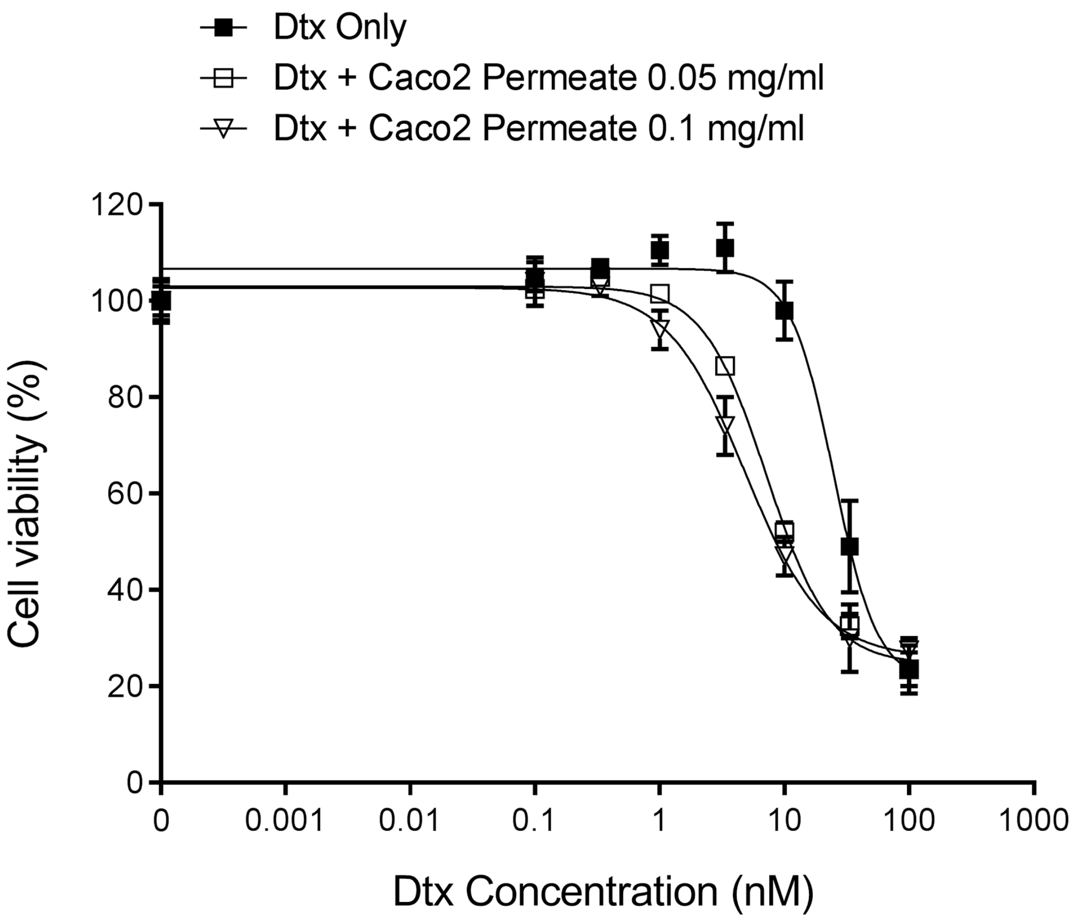

Supplementary Figure S2: The cell viability (measured from triplicate samples) of PC3-TxR cells treated with Dtx in combination with TW Caco-2 permeate collected from the loading concentration of 0.1 and 0.05 mg/ml).

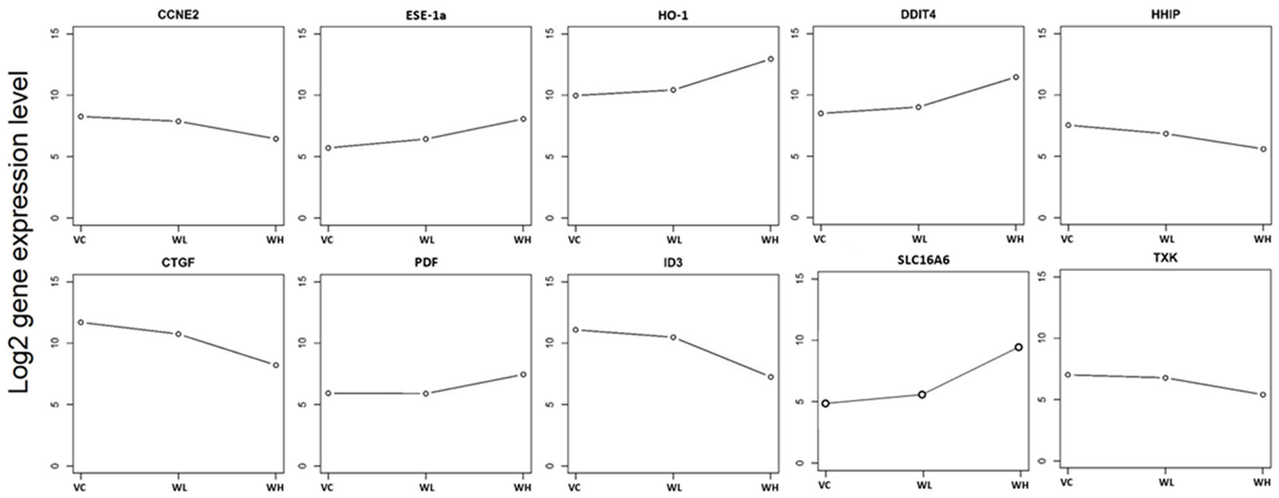

Supplementary Figure S3: Dose dependent DEGs by TW identified in microarray study.

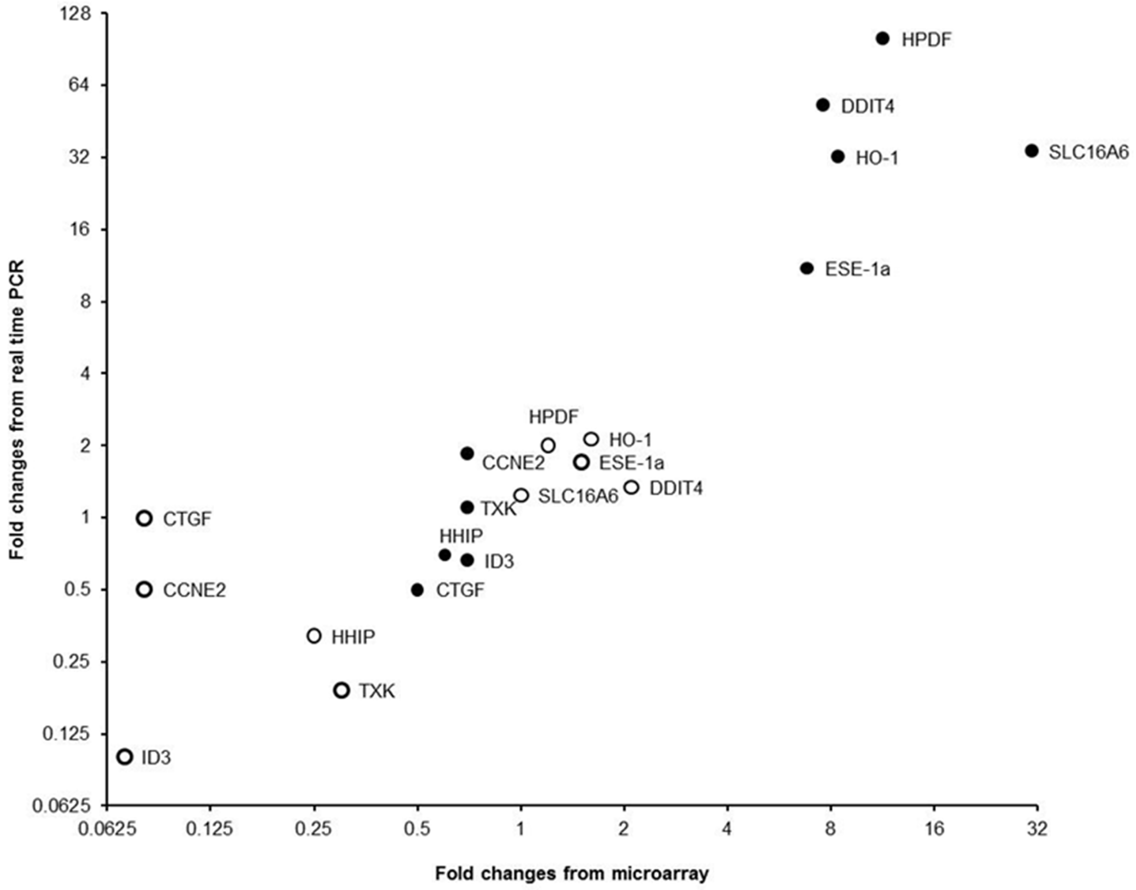

Supplementary Figure S4: Correlation fold changes of ten selected DEGS between microarray and real time PCR approaches (open circle: low dose group, e.g., 0.1 mg/ml TW group; solid circle: high dose group, e.g., 1 mg/ml TW group) (r^2=0.583, P<0.001, Pearson correlation).

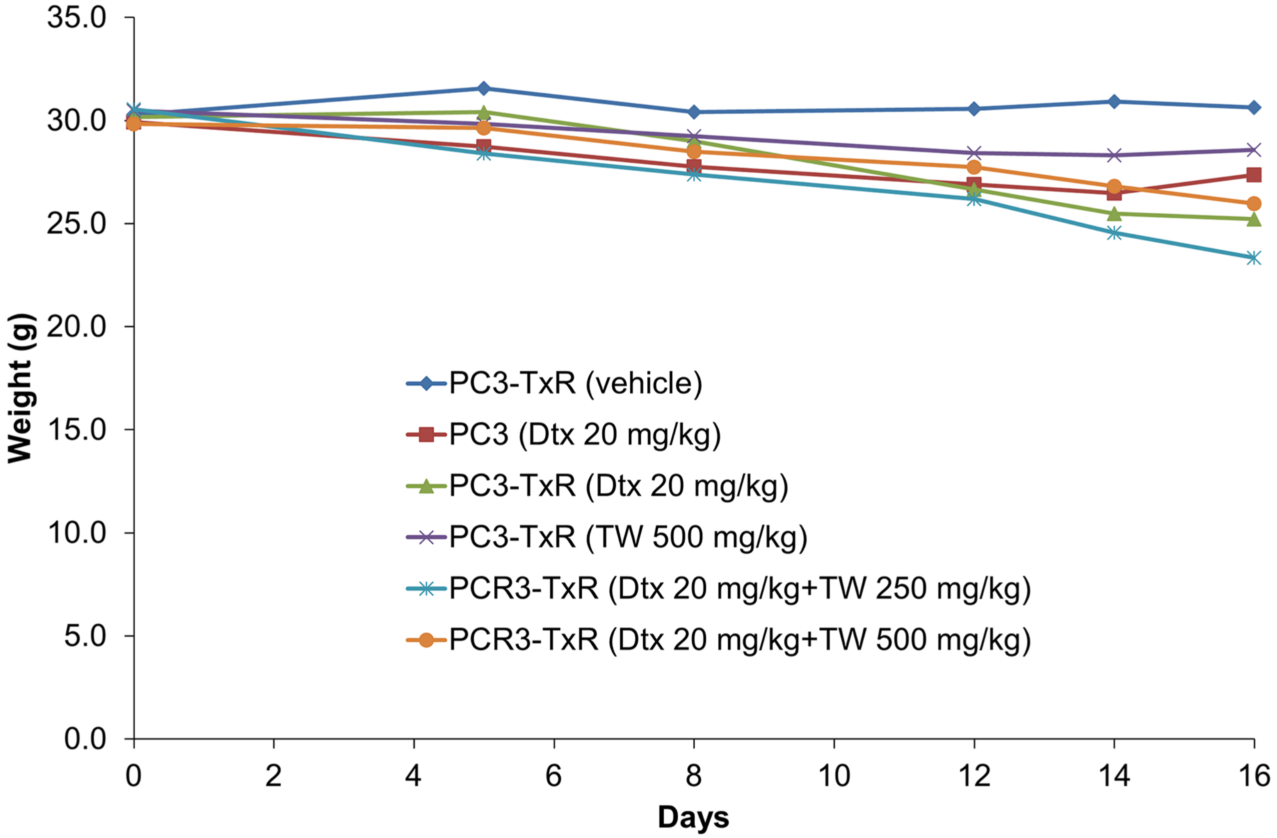

Supplementary Figure S5: Body weight of mice after treatment of docetaxel, TW and their combination.
